# Overexpression of SERPINA3 promotes tumor invasion and migration, epithelial-mesenchymal-transition in triple-negative breast cancer cells

**DOI:** 10.1007/s12282-021-01221-4

**Published:** 2021-02-10

**Authors:** Yingzi Zhang, Jiao Tian, Chi Qu, Yang Peng, Jinwei Lei, Kang Li, Beige Zong, Lu Sun, Shengchun Liu

**Affiliations:** Department of Endocrine Breast Surgery, The First Affiliated Hospital of Chongqing Medical University, Yixueyuan Road, Yuanjiagang, Yuzhong district, Chongqing, China

**Keywords:** Serpin peptidase inhibitor, Clade A (alpha-1-antiproteinase antitrypsin), Member 3 (SERPINA3), Breast cancer, Invasion, Epithelial–mesenchymal transition (EMT), Cisplatin

## Abstract

**Background:**

Recent studies have indicated that serpin peptidase inhibitor, clade A, member 3 (SERPINA3) is a potential marker associated with tumor progression, which connoted that SERPINA3 is related to malignant phenotypes in cancer. However, the biological function of SERPINA3 in breast cancer (BC) remains unclear.

**Methods:**

Bioinformatics data were downloaded from The Cancer Genome Atlas (TCGA) and Gene Expression Omnibus (GEO) databases. Immunohistochemical staining (IHC) was conducted to determine SERPINA3 expression. With strong aggressive abilities, triple-negative breast cancer (TNBC) cell lines (MDA-MB-231, BT549 and MDA-MB-436) were obtained to examine SERPINA3 expression and functions. Wound healing and Transwell assays were performed to measure cell migration and invasion. Cell Counting Kit-8 (CCK-8) assay was conducted to detect cell proliferation abilities and cell viabilities.

**Results:**

SERPINA3 was upregulated in BC tissues. Functional assays suggested that overexpression of SERPINA3 significantly promoted cell proliferation, where migration and invasion of TNBC cells were accelerated. Knockdown of SERPINA3 had the opposite effects. These results causing by overexpression of SERPINA3 were also confirmed in non-TNBC cell lines. Overexpression of SERPINA3 remarkably enhanced the epithelial–mesenchymal transition (EMT) by upregulating the EMT markers and EZH2. In addition, the overexpression of SERPINA3 reduced the sensitivity of TNBC cells to cisplatin.

**Conclusion:**

SERPINA3 can regulate the migration, invasion and EMT of TNBC cells and increased expression of SERPINA3 confers resistance to cisplatin in TNBC cells. We discern it is required for the regulation of BC progression and is a critical target for the clinical treatment of BC.

**Supplementary Information:**

The online version contains supplementary material available at 10.1007/s12282-021-01221-4.

## Introduction

Breast cancer (BC) is the most common malignant tumor among women. In 2018, approximately 2.1 million women were newly diagnosed with BC, and 626,679 women with BC died [1]. The global incidence of BC has been increasing at a rate of 3.1% per year, from 641,000 cases in 1980 to 1.6 million cases in 2010 [2]. The treatment strategies for BC vary by molecular subtype. Currently, four subtypes of BC have been identified: luminal A and luminal B [expressing the estrogen receptor (ER)], basal-like (or triple-negative) and human epidermal growth factor receptor 2 (HER2)-positive. TNBC is the most aggressive type with the highest risk of recurrence among them. At present, new therapeutic targets are urgently needed to treat TNBC [3]. Serpin peptidase inhibitor, clade A (alpha-1-antiproteinase, antitrypsin), member 3 (SERPINA3) is a secreted serine protease inhibitor that inhibits the biological activities of several important serine proteases, such as cathepsin G and chymotrypsin [4], through proteolysis and is also an acute-phase protein that can be stimulated by cytokines [5]. SERPINA3 participates in various biological activities, including inflammation [6], complement activation [7], and regulation of lipid metabolism [8], apoptosis and wound healing [5, 9]. Recently, SERPINA3 has been reported in a variety of solid tumors [9–11]. The high expression of SERPINA3 has been suggested to be associated with tumorigenesis of colorectal carcinomas [10], liver hepatocellular carcinoma (HCC) [12], lung cancer [13, 14], glioblastoma **(**GBM**)** [15] and gastric cancer [16]. For instance, reactive oxygen species (ROS) can induce oxidative modification of specific residues in SERPINA3, a transcriptional activator of PI3Kδ expression in HCC, leading to PI3Kδ signal activation and HCC proliferation [17]. SERPINA3 may promote the progression of invasive GBM cells through the extracellular matrix (ECM) [15]. SERPINA3 also disordered the G2/M checkpoint and inhibited cell apoptosis by activating the mitogen-activated protein kinase (MAPK)/ERK1/2 and PI3K/AKT signaling pathways in endometrial cancer cells [15]. Enhancer of zeste homologue 2 (EZH2), a histone methyltransferase, has been reported to be highly expressed in many human tumors, including BC, and its higher expression has a direct positive correlation with malignant invasion and EMT phenotypes and poor prognosis [18, 19]. EZH2 epigenetically suppresses gene expression through the trimethylation of histone H3 at lysine 27 (H3K27me3) and further mediates cancer cell activities such as proliferation, apoptosis, invasion and migration [20, 21]. However, the role of SERPINA3 in BC and the correlation with aggressive invasion phenotype remains unknown. Therefore, we aimed to investigate the effort of SERPINA3 in the progression of BC and sought to examine the genetic contribution of SERPINA3 to the development and therapy of BC, which might be explored as a potential candidate for clinical treatment.

## Materials and methods

### Patients and samples

A total of 40 paired human breast tissue specimens, including tumor and adjacent normal tissues, were collected from patients at the First Affiliated Hospital of Chongqing Medical University. All patients were diagnosed specifically with BC for the first time by the Clinical Diagnostic Pathology Center of Chongqing Medical University and underwent surgical resection of the BC at the First Affiliated Hospital of Chongqing Medical University. The estrogen receptor (ER) and progesterone receptor (PR) status of the patients were determined according to the results of immunohistochemistry (IHC) by the Clinical Diagnostic Pathology Center of Chongqing Medical University. The study was approved by the Ethics Committee of Chongqing Medical University. Written informed consent was obtained from all patients. All specimens were soaked in liquid nitrogen for preservation immediately after an operation and stored at − 80 °C in a freezer for use in later experiments.

### Bioinformatics analysis

Data showing SERPINA3 expression in BC tissues and normal tissues were downloaded from the Gene Expression Omnibus database (https://cancerge-nome.nih.gov) (GEO42568). SERPINA3 expression in different subtypes of BC cells was determined using Gene Expression Profling Interactive Analysis (GEPIA; http://gepia.cancer-pku.cn/index.html) and Starbase (starbase.sysu.edu.cn/). The results of different assays were used to draw figures via GraphPad Prism 7.0. According to the SERPINA3 gene expression signature, information about four BC subtypes (luminal A, luminal B, HER2-positive and triple-negative) was classified. For survival analysis, clinical data related to invasive breast carcinoma were downloaded from The Cancer Genome Atlas (TCGA) database.

### Reagents

Antibodies against SERPINA3 (cat. no. 66078-1-Ig), GAPDH (cat. no. 60004-1-Ig), Twist1 (cat. no. 25465-I-Ig), Snail (cat. no. 13099- I-Ap), EZH2 (cat. no. 21800-1-AP), ZEB1 (cat. no. 66279-I-Ig), Vimentin (cat. no. 60330-I-Ig) and HRP-conjugated Affinipure goat anti-rabbit IgG (H + L) (cat. no. SA00001-2), HRP-conjugated Affinipure goat anti-mouse IgG (H + L) (cat. no. SA00001-1) secondary antibodies were sourced from Proteintech (Wuhan, China). The Annexin V-FITC/PI Apoptosis Detection Kit (cat. no. FXP018) for flow cytometry analysis was obtained from 4A Biotech Co., Ltd. (Beijing, China), and a cell cycle detection kit (cat. no. C1052) was obtained from Beyotime Biotech Inc. An IHC staining kit (cat. no. SV0004) was obtained from Boster Biological Technology Co., Ltd. Diaminobenzidine (DAB) for IHC (cat. no. PV-9000; dilution 20:1) was purchased from Beijing Zhongshan Golden Bridge Biotechnology Co., Ltd. (OriGene Technologies). Transwell chambers with 8 µm pores and Matrigel were purchased from Corning (Corning, NY).

### Recombinant plasmid overexpression, lentiviral shRNA cloning

The CDS fragment of SERPINA3 was amplified by PCR with primers [Sangon Biotech (Shanghai) Co., Ltd.]C. The PCR products were purified and inserted into the XhoI site of the pcDNA3.1(-) expression vector to obtain the overexpression recombinant plasmid of SERPINA3. The plasmid was confirmed by sequencing from Sangon Biotech (Shanghai) and transfected into MCF-7/T-47D/MDA-MB-231/MDA-MB-436/BT-549 cells using the LipoFilter DNA Transfection Reagent (Thermo Fisher Scientific) according to the manufacturer's instructions. The hU6-MCS-CBh-gcGFP-IRES-puromycin lentiviral shRNA plasmid was created by GeneChem (Shanghai, China). Viral knockdown infections of SERPINA3 with shRNA were performed according to the manufacturer's instructions.

### Cell culture

The human triple-negative breast cancer cells (BT-549, MDA-MB-231, MDA-MB-436 and MDA-MB-468) and HR-positive breast cancer cells (MCF-7 and T-47D) were purchased from the Cell Bank of Shanghai Institute of Biological Sciences, Chinese Academy of Science and maintained in RPMI-Dulbecco’s modified Eagle medium (DMEM)/high glucose medium or RPMI 1640 medium (Gibco; Thermo Fisher Scientific, Inc.) supplemented with 10% fetal bovine serum (FBS) (Invitrogen; Thermo Fisher Scientific, Inc.) in a humidified incubator with 5% CO_2_ at 37 °C.

### Immunohistochemistry (IHC) analysis

The extracted human tissues were fixed with 4% formaldehyde buffer. Deparaffinized specimens were then sectioned into 4-µm-thick slices. Tissue slices were incubated at 60 °C for 2 h before dewaxing, and the slices were autoclaved at 115 °C for 3 min for antigen retrieval in a citric acid buffer (pH 6.0) and quenched for endogenous peroxidase activity with 0.3% H2O2 solution for 15 min. Then, the slices were blocked for nonspecific binding with normal goat serum for 45 min and incubated with the specific primary antibody against SERPINA3 (dilution 1:200) overnight at 4 °C. Subsequently, the sections were treated with the goat anti-mouse secondary antibody for 30 min at room temperature. Protein expression was visualized by 3,3′-diaminobenzidine (DAB). Images were captured using a Nikon Eclipse 80i microscope (Nikon Corporation). According to the expression of SERPINA3 in IHC, we preliminarily analyzed the prognosis of clinical specimens. Criteria of IHC staining intensity was the following:‘−’ (negative) (no or less than 5% positive cells), ‘+’ (5–25% positive cells), ‘ + + ’ (26–50% positive cells), and ‘ + + + ’ (more than 50% positive cells).

### RNA isolation, reverse-transcription reaction and quantitative real-time PCR (qPCR)

Total RNA from cultured cells was extracted with a total RNA extraction kit (Promega Co., Ltd) and reverse-transcribed using the PrimeScript RT Reagent Kit (MedChemExpress Co., Ltd). Quantitative RT-PCR was performed using SYBR Premix Ex TaqTM II (MedChemExpress Co., Ltd) in a 10-µl PCR mixture on a Bio-Rad CFX96 Real-Time PCR system (Bio-Rad Laboratories, Inc.) according to the manufacturer’s instructions. An initial cycling for 2 min at 95 °C, followed by 39 cycles at 95 °C for 30 s, 30 s at 58 °C and 20 s at 72 °C. Primer sequences are listed in Table 1. Three independent experiments were performed for each group. Relative gene expression was normalized to GAPDH and evaluated using the 2^−ΔΔCt^ method.

**Table 1:** Primer sequences used for PCR or constructions of various plasmids

Primer	Sequence (5′–3′)
GAPDH	F: CTCTGCTCCTCCTGTTCGAC
	R: GCGCCCAATACGACCAAATC
SERPINA3 overexpression	F: CTGTCCTCTGCCACCCTA
	R: GGCTGAAAGCGAAGTCC
SERPINA3 real-time PCR	F: TGCCAGCGCACTCTTCATC
	R: TGTCGTTCAGGTTATAGTCCCTC
E-cadherin	F: AGGCCAAGCAGCAGTACATT
	R: CATTCACATCCAGCACATCC
N-cadherin	F: TTTGATGGAGGTCTCCTAACACC
	R: ACGTTTAACACGTTGGAAATGTG
Twist1	F: AGCTACGCCTTCTCCGTCT
	R: TCCTTCTCTGGAAACAATGAC
Snail	F: CGGAAGCCTAACTACAGCGA
	R: GGACAGAGTCCCAGATGAGC
Vimentin	F: GAGAACTTTGCCGTTGAAGC
	R: GCTTCCTGTAGGTGGCAATC
STAT3	F: CAGCAGCTTGACACACGGTA
	R: AAACACCAAAGTGGCATGTGA
ZEB1	F: GATGACCTGCCAACAGACCA
	R: CCCCAGGATTTCTTGCCCTT
EZH2	F: CCCTGACCTCTGTCTTACTTGTGGA
	R: ACGTCAGATGGTGCCAGCAAT

### Western blot analysis

Cells were harvested after being washed twice with cold PBS (4 °C). Protein lysates were prepared using RIPA lysis buffer (Beyotime, China) including protease inhibitor cocktail (EDTA-free, mini-tablet) (Cat. No. HY-K0011, MedChemExpress Co., Ltd), and the protein concentration was measured using the BCA Assay Kit (Beyotime Institute of Biotechnology). Extracted proteins (30 µg/10 µl/lane) from each group were separated by 10% sodium dodecyl sulfate–polyacrylamide gel electrophoresis (SDS-PAGE) (Bio-Rad Laboratories, Inc.). After blocking with 5% non-milk for 70 min at room temperature, the membranes were incubated with specific primary antibodies at 4 °C overnight. Then, after washing three times with Tris-buffered saline containing Tween-20 (TBST), the membranes were incubated with secondary antibodies for 70 min at room temperature. Proteins were visualized by chemiluminescence using enhanced chemiluminescent substrate (New Cell & Molecular Biotech Co., Ltd). Immunoreactive bands were examined using the ChemiDoc Imaging System (Bio-Rad Laboratories, Inc.). Autoradiograms were quantified by densitometry (Quantity One software; Bio-Rad). The GAPDH antibody was used as a control.

### Cell proliferation assay and cell viability assay

MCF-7, T-47D, BT-549, MDA-MB-231, and MDA-MB-436 cells were seeded in 96-well plates 24 h after transfection. Then, cell growth and cell viability were detected using the Cell Counting Kit-8 (CCK-8; Cat. No. HY-K0301; MedChemExpress Co., Ltd.) according to the manufacturer’s instructions to query the potential effect on the proliferation and drug resistance of breast cancer cells by overexpression of SERPINA3. Ten microliters of CCK-8 were added to each well at different time points and incubated at 37 °C and 5% CO_2_ for 60–90 min. The optical density (OD) was measured at 450 nm using a SynergyH1 microplate reader (BioTek Instruments, Inc.). Empty wells served as blank controls.

### Cell cycle assay and flow cytometry analysis

Cell pellets were harvested 72 h after transfection by centrifugation and washed twice with 4 °C PBS and fixed with 75% ethanol for 24 h at 4 °C. Staining for DNA content was performed in 500 µl of the mixture solution using a cell cycle detection kit for 30 min at 37 °C in a water bath in the dark and was analyzed using flow cytometry with a BD FACS Verse Flow Cytometer. The data were analyzed by FlowJo software.

### Wound healing assays

Cells were seeded in 6-well plates. When cells grew in a full monolayer, a wound was produced by scraping across the cell monolayer using a 10 μl sterile tip and gently washed with PBS. Then, the medium was replaced with serum-free DMEM, and photographs were taken immediately (0 h). The distance migrated by the cell monolayer to close the wounded area during this time period was measured to reveal the aggressive phenotype and the migration promoting feasibility of SERPINA3 in breast cancer. All experiments were repeated at least 3 times.

### Transwell assays

A total of 80,000 cells in 300 µl serum-free medium were seeded into the upper chamber of a Transwell plate, and 600 µl complete medium was added to the lower compartment. The cells were incubated for 8 h in a humidified incubator with 5% CO_2_ at 37 °C, and then the upper chamber was wiped twice with cotton swabs. Next, cells were fixed with 4% paraformaldehyde for 15–20 min and stained with 0.5% crystal violet for another 15–20 min at room temperature. The numbers of invading cells in five randomly selected fields were counted under an inverted light microscope (magnification, × 200; TE2000-U; Nikon Corporation). Three replicates were performed, which can be well applied to demonstrate the invasion ability of breast cancer cells under the interfering of SERPINA3.

### Statistical analysis

All experiments were independently performed at least three times. The mean ± standard deviation (SD) was determined for each group. Statistical analyses were performed using one-way analysis of variance (ANOVA) for multiple group comparisons or Student’s *t*-test for individual comparisons. *P* < 0.05 was considered statistically significant.

## Results

### Upregulated expression of SERPINA3 in BC

**Fig. 1:**
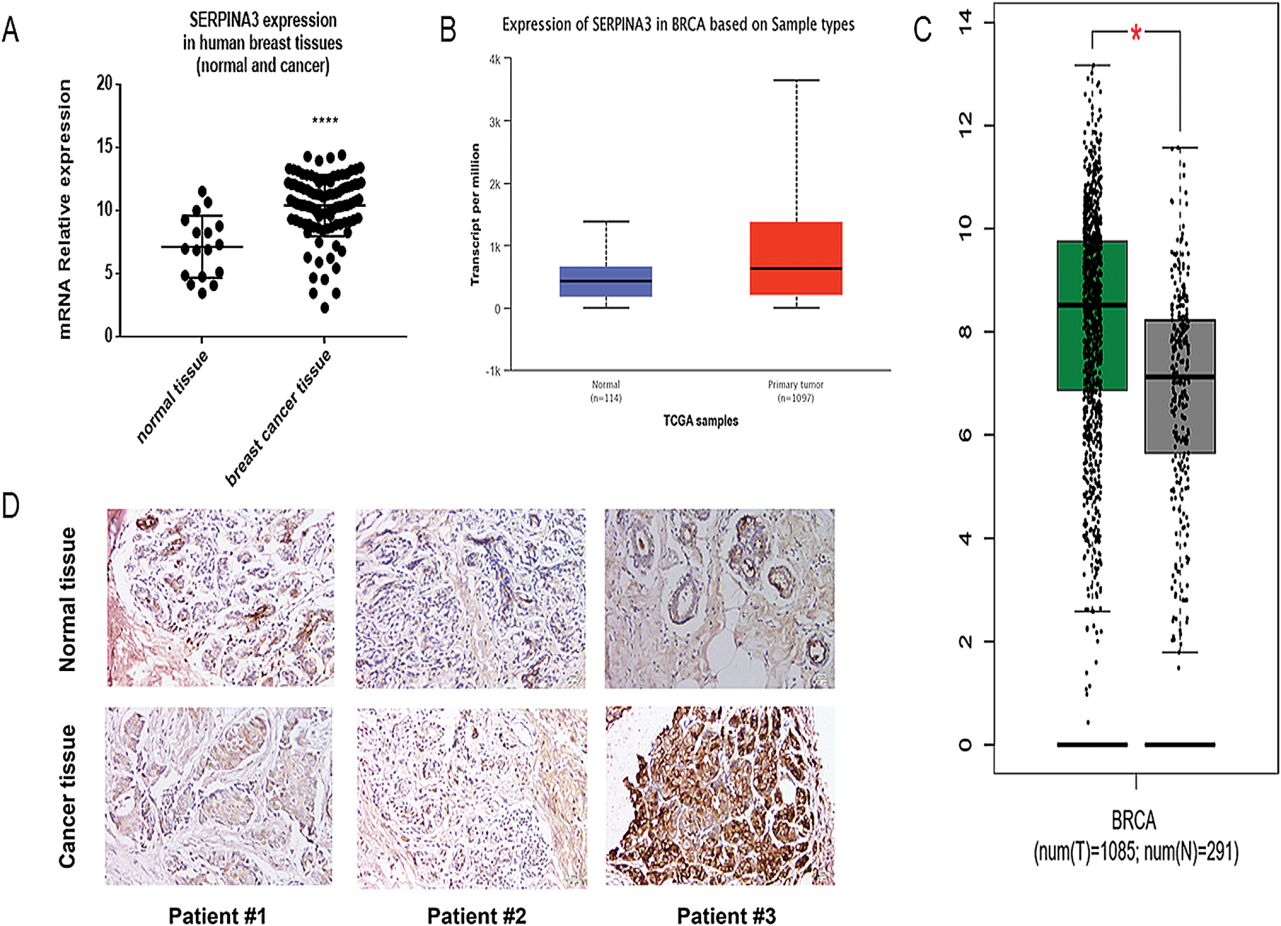
Overexpression of SERPINA3 is associated with breast cancer tumorigenesis. **a–c** Gene expression data from GEO database, TCGA database and GEPIA database showed that *SERPINA3* mRNA level was overexpressed in human breast cancer (The values are compared to a normal group and are represented as the means ± S.E.M., *P < 0.05; **P < 0.01; ***P < 0.001; ****P < 0.0001). **d** IHC staining of SERPINA3 expression in normal and breast cancer tissues (100 × ; 400 ×), mostly located in the nucleus. The staining of SERPINA3 in the liver was considered as a positive control (100 ×; 400 ×)

We examined BC databases from the TCGA, GEO and Gene Expression Profling Interactive Analysis (GEPIA) to evaluate the differential expression of SERPINA3 between BC and normal tissues. Analysis of the GEO database indicated that cancer samples with SERPINA3 transcripts (*n* = 104) had a significantly higher expression than normal samples (*n* = 17) (*P* < 0.0001) (Fig. 1a) and this was also shown in data from the TCGA (Fig. 1b, c). Similar results were also found in IHC analysis (Fig. 1d). In the whole experiment, IHC, Western Blot and RT-qPCR experimental techniques were applied to evaluate the expression of SERPINA3 in protein and transcriptional level, respectively, in BC cell lines. As displayed in Fig. 1d, SERPINA3 was mainly expressed in the nucleus of cancer cells and ECM but rarely expressed in normal para-cancer tissues conducting by IHC. We next estimated the effect of SERPINA3 as an oncogenic biomarker for the overall survival of patients diagnosed with BC from the TCGA database, which were divided into two groups according to the differential expression of the SERPINA3 gene. The results indicated that TNBC with higher expression levels of SERPINA3 may correlate with poor prognosis (*P* = 0.58, Online Resource 1a). Univariate and multivariate analyses of clinicopathological characteristics of OS for BC patients are shown in Table 2. Meantime, we performed the disease-free survival analysis referring to the BC patients whose clinical information were collected for IHC and the patient characteristics were shown in Table 3. According to the intrinsic subtypes of the extant patient data, the survival analysis was conducted in TNBC and non-TNBC (HR positive) populations (Online Resource 1b). Since TNBC cell lines had a good tumorigenic effect, we selected MDA-MB-231, MDA-MB-436 and BT-549 cell lines as experimental models.

**Table 2:** Univariate and multivariate analyses of clinicopathological characteristics of OS

	Univariate analysis	*P* value	Multivariate analysis	*P* value
	HR (95% CI)		HR (95% CI)	
SERPINA3	0.957 (0.924–0.991)	0.015	0.967 (0.923–1.013)	0.16
Laterality	0.963 (0.847–1.094)	0.563		
Surgery	1.618 (1.412–1.854)	0.001	1.507 (1.291–1.757)	0.001
ER Status	0.917 (0.787–1.068)	0.266		
HER2 Status	1.412 (1.17–1.703)	0.001	1.28 (1.023–1.6)	0.031
PR Status	0.822 (0.724–0.934)	0.003	0.905 (0.767–1.069)	0.241
Tumor Stage	2.241 (1.785–2.813)	0.001	1.95 (1.544–2.463)	0.001

Comparisons were considered statistically significant when *P* < 0.05

**Table 3:** Patients characteristics

	Number of patients (*n*)	(%)
Age (years)		
≤ 50	18	45
> 50	22	55
Tumor size (cm)		
≤ 2	7	17.5
> 2	33	82.5
Histological grade		
I	3	7.5
II	24	60
III	11	27.5
Unknown	2	5
Lymph node metastasis		
Positive	25	62.5
Negative	10	25
Unknown	5	12.5
ER status		
Positive	26	65
Negative	14	35
PR status		
Positive	20	50
Negative	20	50
HER2 status		
Positive	32	80
Negative	8	20
Ki-67 (%)		
≤ 30	21	52.5
> 30	19	47.5
Molecular subtypes		
Luminal A-like	5	12.5
Luminal B-like	22	55
HER2 positive	10	25
Triple negative	3	7.5
Adjuvant therapy		
Yes	19	47.5
No	21	52.5

*ER* estrogen receptor, *PR* progesterone receptor, *HER2* human epidermal growth factor receptor 2

### SERPINA3 promoted TNBC cell proliferation

**Fig. 2:**
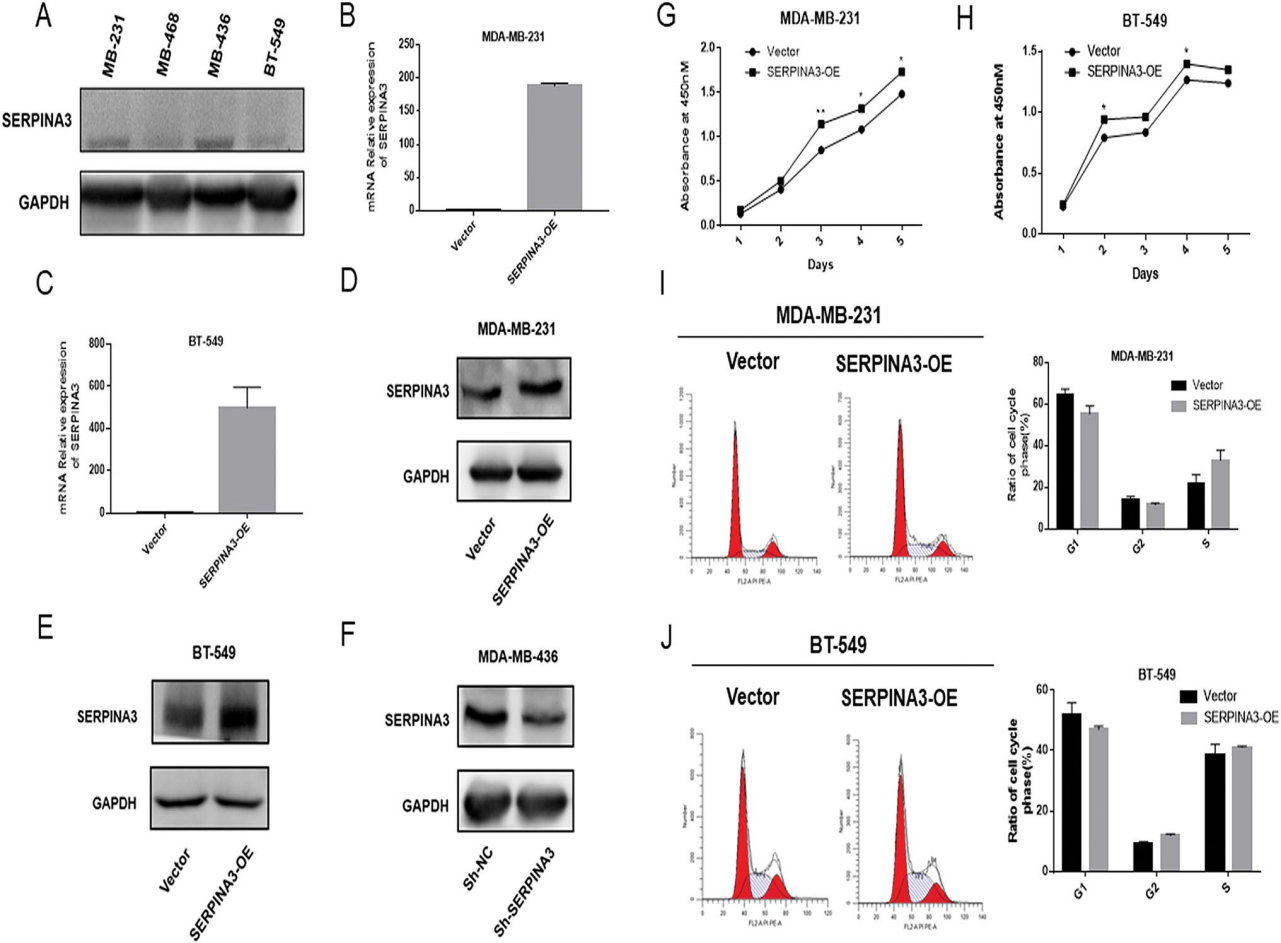
Confirmation of overexpression and interference of SERPINA3 and SERPINA3 influenced on TNBC cells proliferation. **a** The protein level of SERPINA3 in triple-negative breast cancer cell lines: MDA-MB-231, MDA-MB-436, MDA-MB-468, and BT-549. **b–e** Real-time PCR and Western blot revealed the expression of SERPINA3 mRNA and protein was efficiently overexpressed in MDA-MB-231 and BT-549 cells. **f** Western blot revealed the expression of SERPINA3 protein was efficiently inhibited in MDA-MB-436 cells. **g, h** Overexpression of SERPINA3 promoted TNBC cell proliferation. The growth curve of MDA-MB-231 and BT-549 cells infection of SERPINA3-vector and SERPINA3-OE was displayed. Viability of SERPINA3-OE targeted cells and control cells were observed for 5 days. (*P < 0.05; **P < 0.01; ***P < 0.001; ****P < 0.0001). **i**, **j** SERPINA3 overexpression induced S-phase cell cycle arrest. Flow cytometric assay was employed to analyze the cell cycle distribution. The percentage of cells in the S phase was evidently increased, compared with the control group

**Fig. 3:**
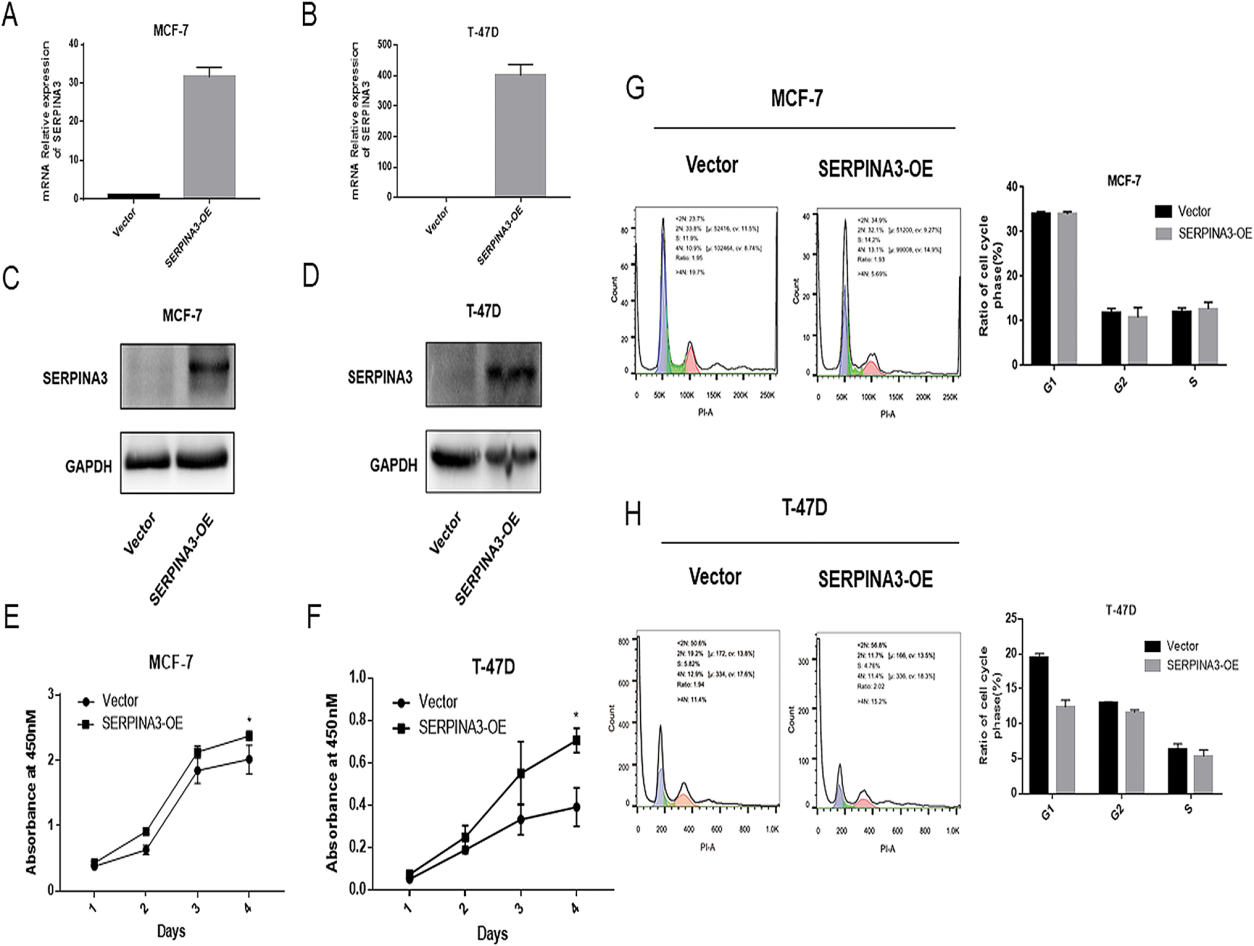
Validation of overexpression of SERPINA3 and SERPINA3 influenced on non-TNBC cells proliferation. **a–d** Real-time PCR and Western blot revealed the expression of SERPINA3 mRNA and protein was efficiently overexpressed in MCF-7 and T-47D cells. **e**, **f** Overexpression of SERPINA3 also promoted non-TNBC cell proliferation. The growth curve of MCF-7 and T-47D cells infection of SERPINA3-vector and SERPINA3-OE was displayed. Viability of SERPINA3-OE targeted cells and control cells were observed for 4 days. (*P < 0.05; **P < 0.01; ***P < 0.001; ****P < 0.0001). **g**, **h** SERPINA3 overexpression hardly influenced the cell cycle in MCF-7 and T-47D cells. Flow cytometric assay was employed to analyze the cell cycle distribution

The aberrant expression of SERPINA3 indicated that SERPINA3 might play a crucial role in the carcinogenesis of BC. To confirm the overexpression of the SERPINA3 gene in TNBC cells, we utilized a series of human TNBC cell lines (MDA-MB-231, MDA-MB-436, MDA-MB-468, and BT-549) to examine SERPINA3 protein expression by western blotting (Fig. 2a). To investigate the biological function of SERPINA3 in BC cells, we constructed a plasmid overexpressing SERPINA3 and synthetized lentivirus with shRNA targeting SERPINA3 (shSERPINA3). The overexpression and interference efficiencies of SERPINA3 in MDA-MB-231, MDA-MB-436 and BT-549 cells were confirmed by qPCR and western blotting, respectively. The results showed that the transcriptional level of SERPINA3 was increased in MDA-MB-231 cells, BT-549 cells (Fig. 2b, 2c) and MDA-MB-436 cells (Online Resource 2). Since the background gene expression in unequal cells was different, the level of overexpression differed so that the highly SERPINA3-expressing MDA-MB-436 TNBC cell line was selected to conduct the follow-up knockdown assays. Similarly, we also verified the overexpression of SERPINA3 at the protein level (Fig. 2d, e) and the inhibition in MDA-MB-436 cells (Fig. 2f). Consequently, to analyze the effect of SERPINA3 on the proliferation of TNBC cells, BT-549 cells and MDA-MB-231 cells were transfected with pcDNA3.1-SERPINA3 and pcDNA3.1 (as controls), respectively. The overexpression of SERPINA3 promoted cell proliferation (Fig. 2g, h). To further elucidate the effect of SERPINA3 on BC growth, we analyzed the cell cycle distributions of SERPINA3-overexpression (SERPINA3-OE) in BT-549, MDA-MB-231 and MDA-MB-436 cells by flow cytometry analysis, and the results indicated that cell proliferation was blocked at G1 in the experimental group and that the number of cells in S phase increased (Fig. 2i, j). However, no significant effect on apoptosis was observed (Online Resource 3a, 3b). Together, these data suggested that the overexpression of SERPINA3 can promote TNBC cell proliferation. The similar results were obtained in non-TNBC cell lines in the same way, including MCF-7 and T-47D. The expression of SERPINA3 after transfecting the overexpression plasmid was increased, shown in Fig. 3a–d. After 4 days of observation, the capability of SERPINA3 to promote th growth of non-TNBC cells was also verified (Fig. 3e, f). Howbeit, the cell cycle was surprisingly found to be unaffected significantly (Fig. 3g, h) and the phenotype of apoptosis in MCF-7 and T-47D was consistent with the observation in TNBC cell lines (Online Resource 3c, 3d).

### SERPINA3 overexpression enhanced the invasion and migration of TNBC cells

**Fig. 4:**
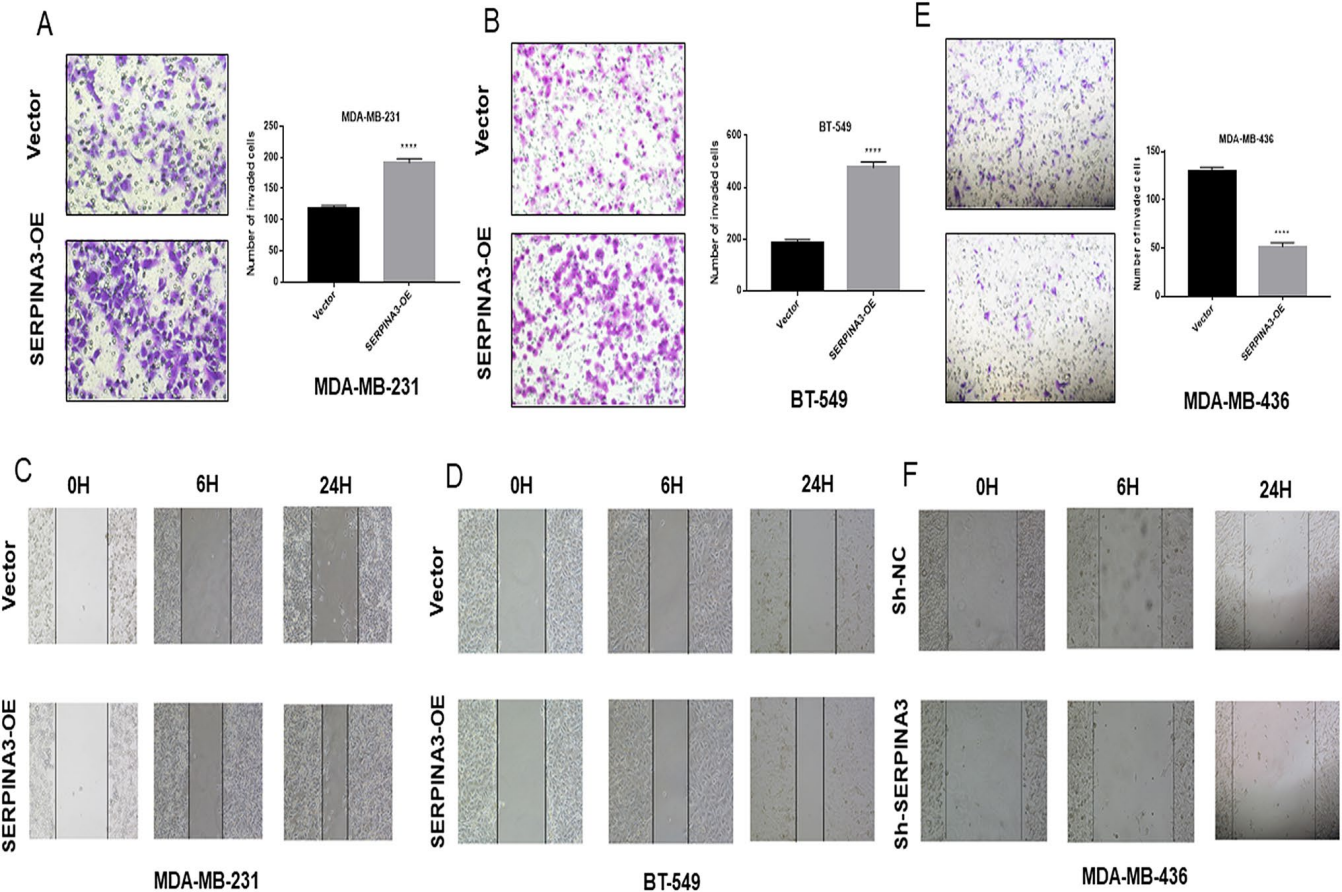
SERPINA3 influenced TNBC cells migration and invasion. **a**, **b** Cell invasions were detected using transwell assay in MDA-MB-231 and BT-549 cells with overexpression of SERPINA3. Values are shown as means ± S.E.M (*n* = 3 per group) (*P < 0.05; **P < 0.01; ***P < 0.001; ****P < 0.0001). **c**, **d** Cell migrations were detected using wound healing assay in MDA-MB-231 and BT-549 cells with overexpression of SERPINA3. Values are shown as means ± S.E.M (*n* = 3 per group) (*P < 0.05; **P < 0.01; ***P < 0.001; ****P < 0.0001). **e**, **f** Cell migration and invasion were detected using wound healing assay and transwell assay in MDA-MB-436 cells with inhibition of SERPINA3. Values are shown as means ± S.E.M (*n* = 3 per group) (*P < 0.05; **P < 0.01; ***P < 0.001; ****P < 0.0001)

We next determined whether SERPINA3 promotes breast stromal tumor cell invasion. We performed transwell assays, which showed that the overexpression of SERPINA3 caused an increase in cell invasion in TNBC cell lines (Fig. 4a, b; Online Resource 4). The wound-healing assay also confirmed that the overexpression of SERPINA3 narrowed the scratch area to promote the migration in TNBC cell lines (Fig. 4c, d; Online Resource 4). Conversely, knockdown of SERPINA3 inhibited the migration and invasion of MDA-MB-436 cells, which was concordant with the experiments with the overexpression of SERPINA3 (Fig. 4e, f). These results suggested that SERPINA3 promotes the invasion and migration of TNBC cells.

**Fig. 5:**
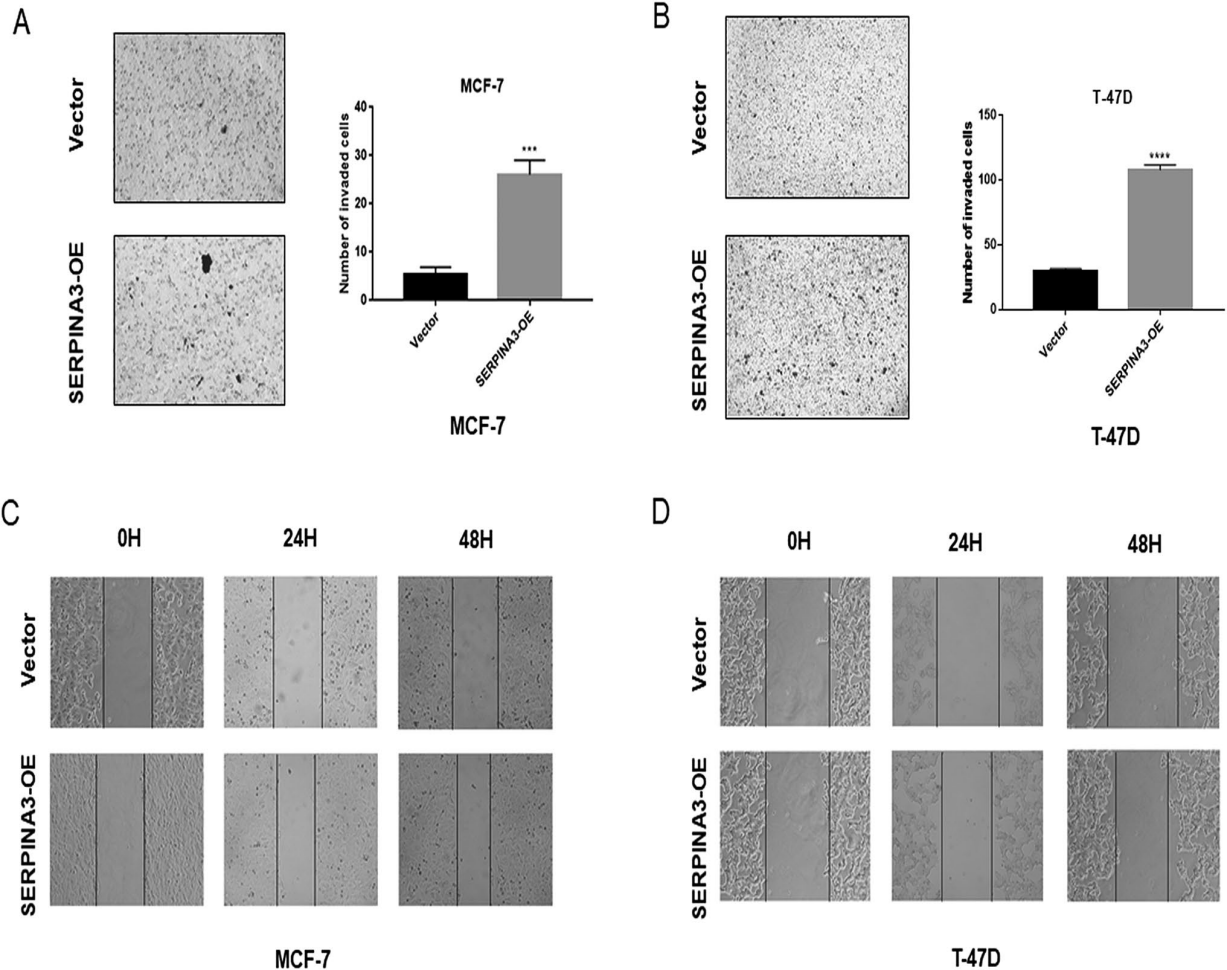
SERPINA3 influenced migration and invasion of non-TNBC cells. **a**, **b** Cell invasions were detected using transwell assay in MCF-7 and T-47D cells with overexpression of SERPINA3. Values are shown as means ± S.E.M (*n* = 3 per group) (*P < 0.05; **P < 0.01; ***P < 0.001; ****P < 0.0001). **c**, **d** Cell migrations were detected using wound healing assay in MCF-7 and T-47D cells with overexpression of SERPINA3. Values are shown as means ± S.E.M (*n* = 3 per group) (*P < 0.05; **P < 0.01; ***P < 0.001; ****P < 0.0001)

This phenotype was verified in non-TNBC cell lines (MCF-7 and T-47D), although the invasive ability of HR-positive BC cells was remarkably lower than that of TNBC cells, shown in Fig. 5a–d.

### SERPINA3 overexpression promoted epithelial–mesenchymal transition (EMT) in TNBC cells

**Fig. 6:**
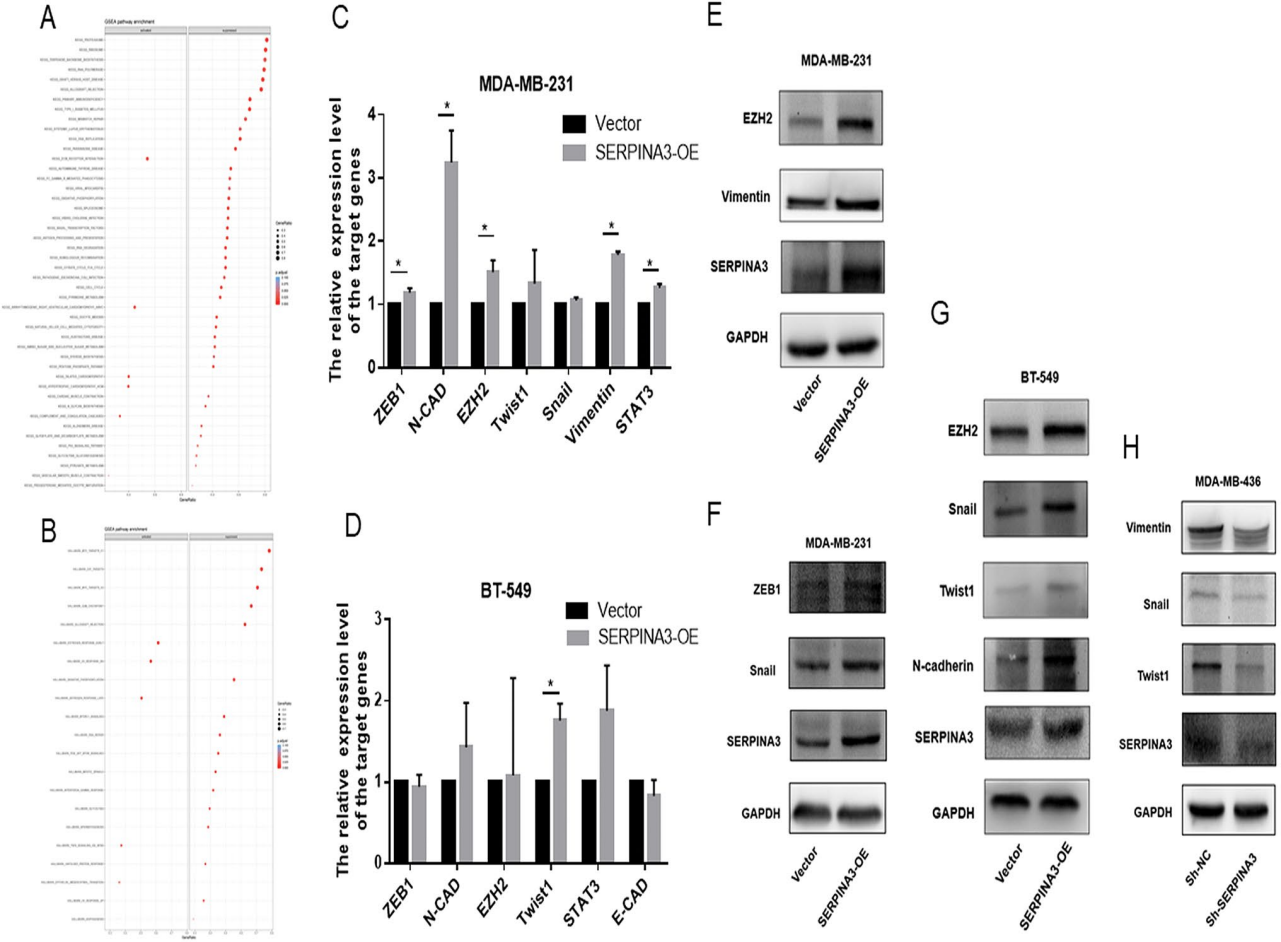
SERPINA3 overexpression promoted epithelial–mesenchymal transition (EMT) in TNBC cells. **a**, **b** Data from KEGG and HALL marker databases revealed the downstream potential pathways influenced by SERPINA3 expression, which strongly suggested that ECM-related pathway and EMT were remarkably activated. **c**, **d** EMT marker E-cadherin, N-cadherin, Vimentin, ZEB1, snail, Twist1 and STAT3 mRNA expression in MDA-MB-231 and BT-549 cells with SERPINA3-OE, compared with the control group (*P < 0.05). **e–g** EMT marker Vimentin, ZEB1, snail, Twist1, N-cadherin and EZH2 protein expression in MDA-MB-231 and BT-549 cells with SERPINA3-OE, compared with the control group. **h** EMT marker Vimentin, snail and Twist1 protein expression in MDA-MB-436 cells with SERPINA3-shRNA, compared with the control group

**Fig. 7:**
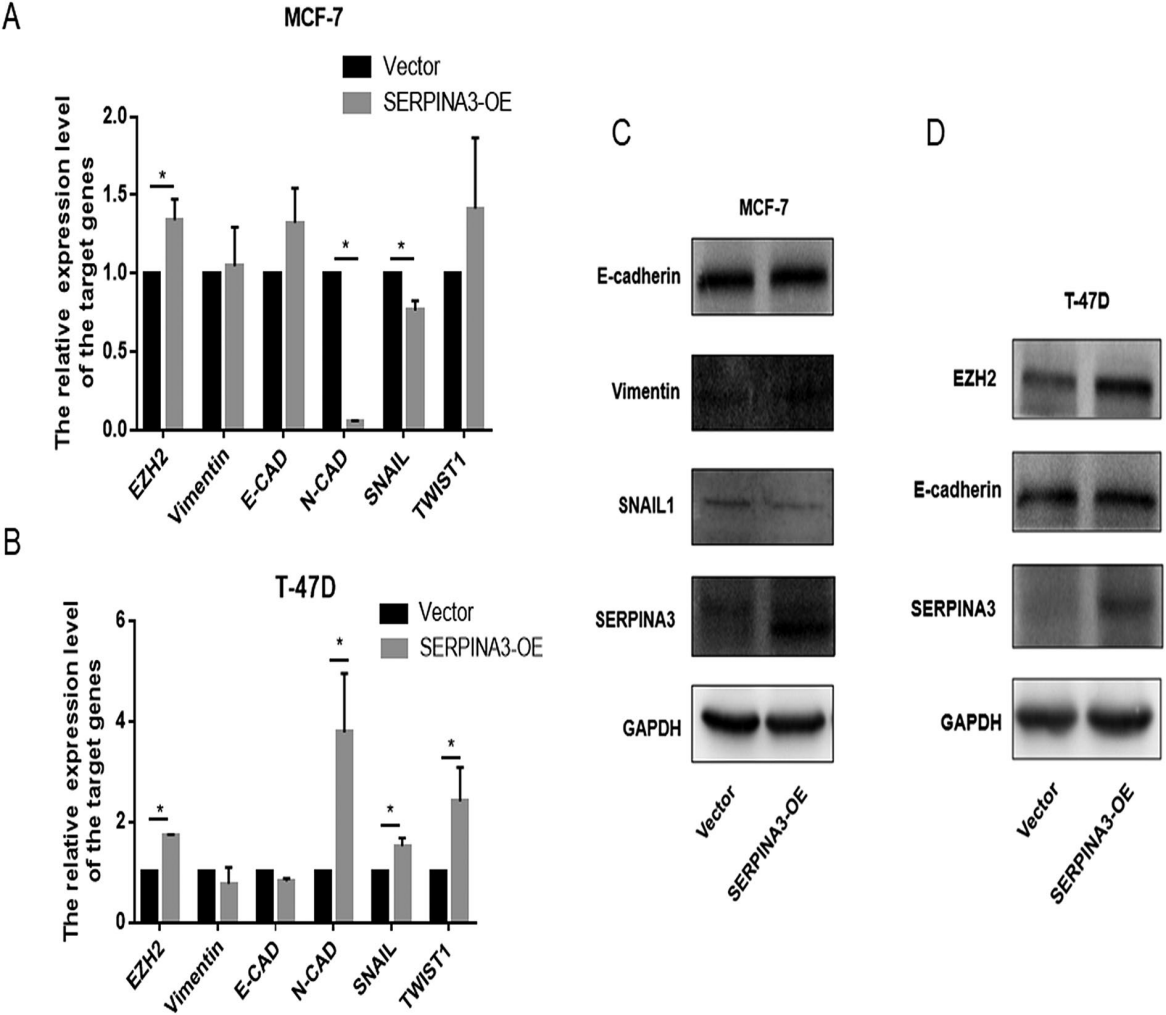
The influence of SERPINA3 overexpression on epithelial–mesenchymal transition (EMT) in non-TNBC cells. **a**, **b** EMT marker E-cadherin, N-cadherin, Vimentin, snail, and Twist1 mRNA expression in MCF-7 and T-47D cells with SERPINA3-OE, compared with the control group (*P < 0.05). **c**, **d** EMT marker E-cadherin, Vimentin, snail and EZH2 protein expression in MCF-7 and T-47D cells with SERPINA3-OE, compared with the control group

Since the promotion of SERPINA3 in BC was confirmed, we investigated the pathways related to BC and molecular functions in different databases. We discovered that ECM-receptor interactions and EMT were obviously activated, as shown in the Kyoto Encyclopedia of Genes and Genomes (KEGG) and HALL marker databases (Fig. 6a, b). It is well known that the epithelial–mesenchymal transition (EMT) process is an important mechanism by which cancer cells become capable of invasion and metastasis [22–25]. Since SERPINA3 can promote breast stromal tumor cell invasion, we further investigated whether SERPINA3 interferes with the cellular phenotype switching of breast stromal tumor cells by measuring the expression of EMT cellular biomarkers, including E-cadherin, N-cadherin, vimentin, Snai1, Twist1, and ZEB1. As shown in Fig. 6c, d, the SERPINA3-OE group treatment significantly upregulated the expression of N-cadherin, vimentin, Snai1, and Twist1 but downregulated E-cadherin at the transcriptional level. Additionally, the same results were indicated at the protein level (Fig. 6e–g). In turn, the inhibition of SERPINA3 correspondingly reversed these results (Fig. 6h). Nevertheless, when we attempted to validate this phenomenon in HR-positive cell lines, it did not come out ideally. The expression of corresponding EMT-related molecules differed at transcription and protein levels with the overexpression of SERPINA3 (Fig. 7a–d). These data connoted that overexpression of SERPINA3 can promote triple-negative breast tumor cell invasion by promoting the EMT process.

### Overexpression of SERPINA3 promotes EZH2 expression

**Fig. 8:**
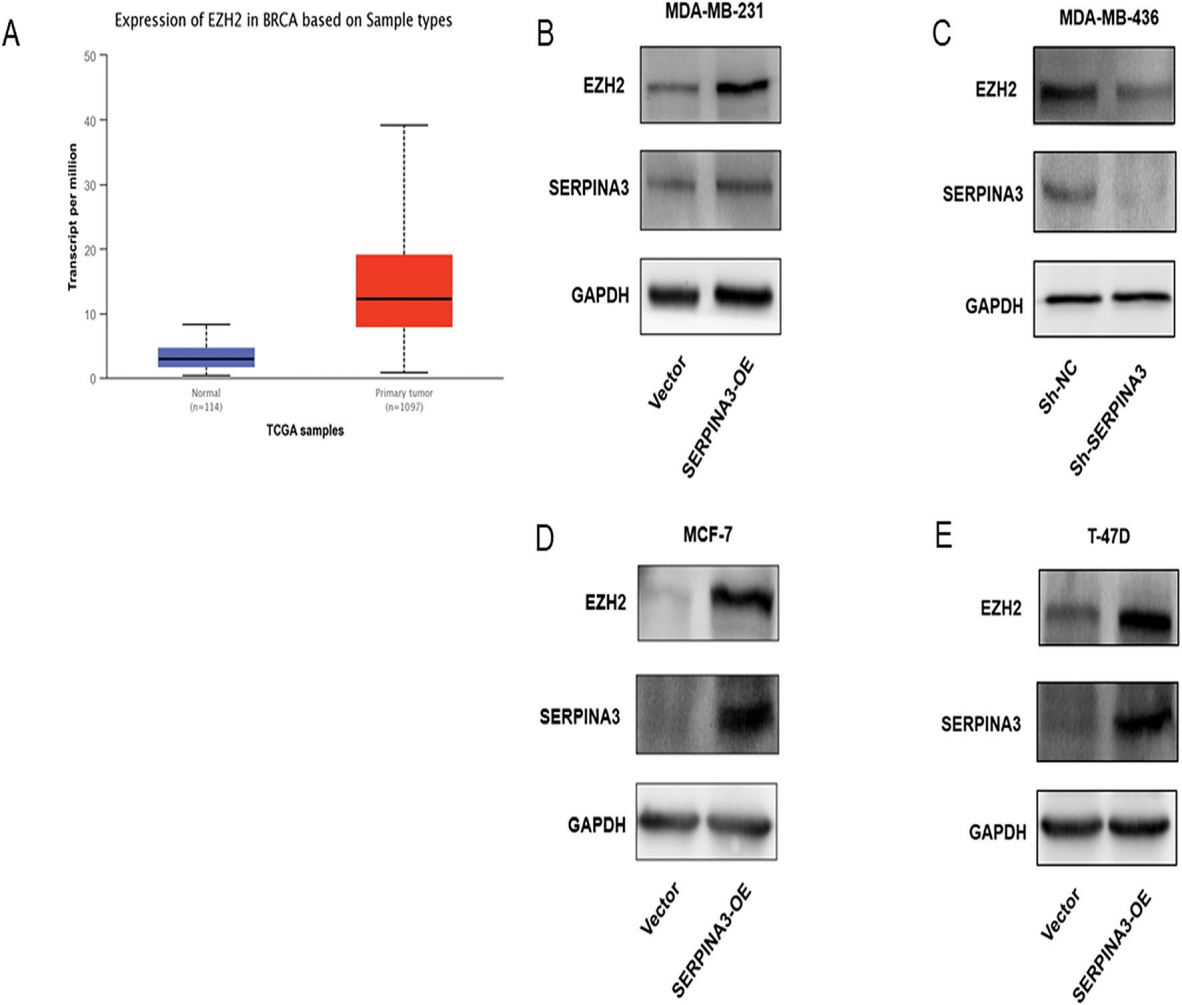
SERPINA3 activated EMT by increasing EZH2 in TNBC cells. **a** EZH2 is significantly highly expressed in BC using the TCGA database analysis. **b**, **c** EZH2 protein expression in MDA-MB-231 and MDA-MB-436 cells with SERPINA3-OE and SERPINA3-shRNA, compared with the control group. **d**, **e** EZH2 protein expression in MCF-7 and T-47D cells with SERPINA3-OE, compared with the control group

Using the TCGA database, we found that EZH2 is significantly highly expressed in BC (Fig. 8a). Various reports have discovered that knockdown of EZH2 induces a mesenchymal to epithelial phenotypic reprogramming and blocks invasion in BC and the unexpected effect of EZH2 in inducing the MAPK signaling pathway was covered, an important regulator of BC invasion and metastasis [26–28]. Of particular interest is that after the overexpression or knockdown of SERPINA3, EZH2 in the TNBC cell lines changed synergistically at the transcription level (Fig. 6c, d) and protein level remarkably (Fig. 8b, c). This appearance was further enhanced in HR-positive BC cells at mRNA and protein levels (Figs. 7a, b, 8d, e). The findings strongly demonstrated that the overexpression of SERPINA3 upregulated the expression of EZH2, which may involve in the process of invasion and EMT induced by SERPINA3.

### Effects of SERPINA3 on the drug sensitivity of TNBC cells

**Fig. 9:**
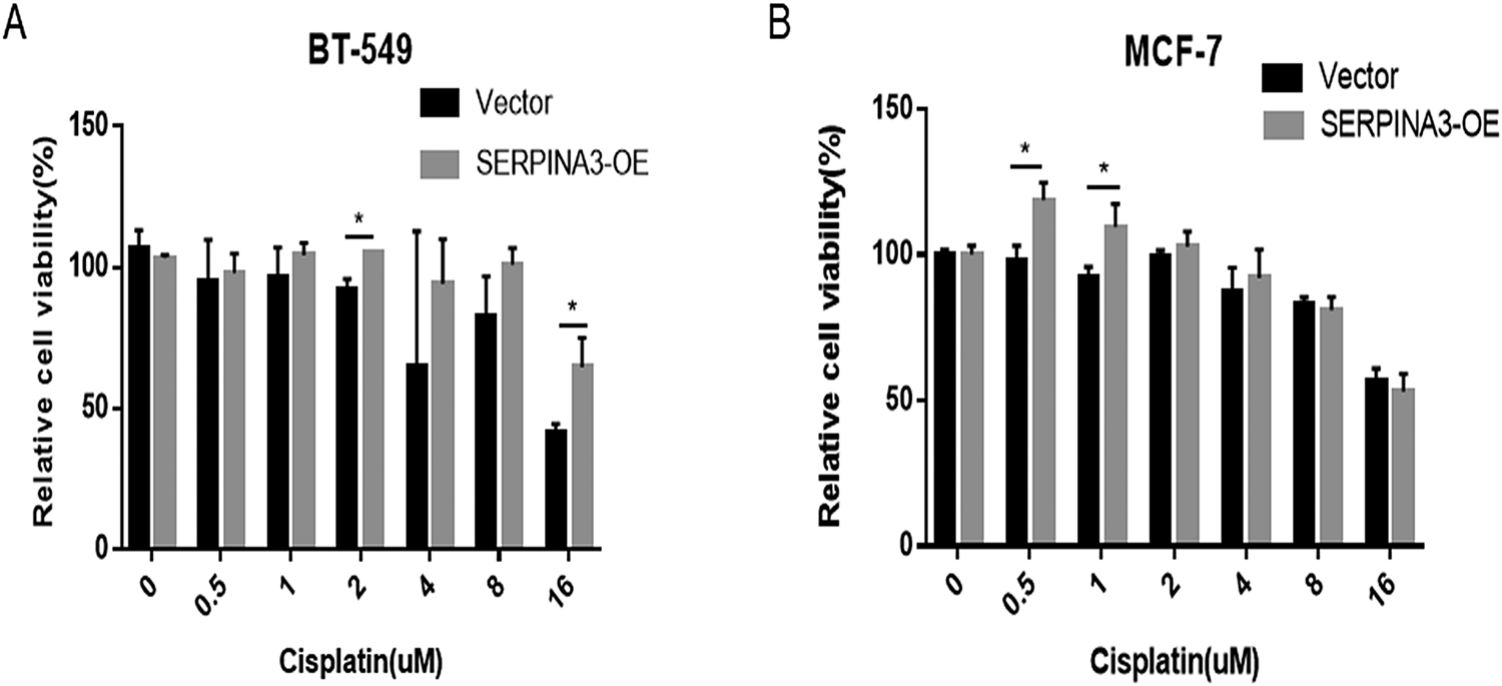
Effects of SERPINA3 on the drug sensitivity of BC cells. **a**, **b** Comparation of cell viability of the SERPINA3-OE group in BT-549 and MCF-7 cells treated with a series of concentrations of cisplatin (μM) for 48 h and the control group (*P < 0.05)

Currently, BC treatment guidelines include surgery, radiotherapy and systemic therapy. We wondered whether the overexpression of SERPINA3 can affect the drug sensitivity of TNBC cells to the abovementioned chemotherapeutics. The overexpression SERPINA3 plasmid was used in the TNBC cell lines BT-549 and MDA-MB-231, and certain concentrations of cisplatin (µM), which were based on IC50 values, were added. The CCK-8 detection method was performed to detect the differences in the sensitivity of the two groups of cells to cisplatin and the changes when SERPINA3 was overexpressed. Compared with the vector (VE) group as a control, the cell viability of the SERPINA3-OE group in BT-549 cells treated with a series of concentrations of cisplatin for 48 h was especially higher than that of the control group (*P* < 0.05, Fig. 9a), while the phenomenon was not observed similarly in MDA-MB-231 cells (Online Resource 5a). These results indicate that overexpression of SERPINA3 can remarkably reduce the sensitivity of BT-549 cells to cisplatin. Delightedly, in HR-positive BC cells, the SERPINA3 overexpression group seemed to own stronger proliferation viability than the control group with the treatment of cisplatin in MCF-7 cells (*P* < 0.05, Fig. 9b), also in T-47D cells (*P* < 0.05, Online Resource 5b). This also implied that SERPINA3 may have a certain effect on cisplatin resistance of breast cancer cells, which need more efforts to go a step further to verify.

## Discussion

BC is one of the deadliest tumors worldwide. It is currently believed that early BC or BC that only spreads to the axillary lymph nodes can be cured. The main treatment goal of advanced BC is to prolong the survival of patients and control the symptoms of low toxicity associated with treatment [3]. It is worth noting that TNBC patients have a high degree of malignancy, a poor prognosis, and limited therapeutic targets, which are essential for finding new diagnostic and prognostic targets and tailored chemotherapy schedules for TNBC patients. As the report goes, several members of the serpin family are related to the immune response, cancer cell growth and apoptosis, cytokine and biological metabolism regulation, cell repair [5–8], etc. Here, we investigated the function of SERPINA3 in BC. The results showed that compared with that in corresponding adjacent non-BC tissues, SERPINA3 was remarkably higher in BC tissues. Nor is this all: SERPINA3 overexpression can induce BC cell proliferation, invasion, migration and EMT. To study the potential mechanism by which SERPINA3 regulates BC invasion and migration, qPCR was used to analyze several important molecules, including Snail, Twist1, ZEB1, and EZH2. Therefore, we found that the transcription level of EMT-related molecular markers increased with the overexpression of SERPINA3 in TNBC cell lines. Among them, EZH2 was first identified as a candidate gene to study further. We found that in pancreatic cancer cells, Twist1 interacts with Ring1B and EZH2 to regulate the expression of E-cadherin and p16 protein, thereby promoting the metastasis of pancreatic cancer [29]. Similarly, in pancreatic cancer, EZH2 promoted EMT and distant metastasis by mediating microRNA-139-5p [30]. We found that EZH2 also changed significantly positively with changes in SERPINA3 at the protein and mRNA level. We suspect that SERPINA3 can interact with EZH2 to induce EMT. However, further work is needed to prove whether SERPINA3 can be directly correlated with EZH2. Thereby effectively reducing the expression of SERPINA3, offering a promising therapeutic strategy for the treatment of aggressive breast cancer cells. SERPINA3 is also an inflammatory protein regulated by cytokines, many of which are of great importance, including interleukin-6 (IL-6) and interleukin-1 (IL-1). Imbalance of cytokines can lead to high or low activation of the immune system, leading to autoimmune diseases or immunosuppression, which is conducive to infection and tumor growth [31]. This is also a possible clinical direction for further research. Considering the relevance between cancer and inflammation, cytokines seem to contribute to tumor growth, as well as effective anti-tumor immunity [32]. We also found that the sensitivity of SERPINA3-overexpressing TNBC cell lines to cisplatin was significantly reduced, which also indicated that SERPINA3 may reduce the sensitivity of BC to cisplatin. This discovery is much pleasant and deserves further confirmation by more drug research, which also inspired us to determine whether the targeted drugs for SERPINA3 can be combined with first-line chemotherapy drugs for BC to further improve the quality of life of TNBC patients. To our knowledge, there is no research reported the expression of SERPINA3 related to prognosis or response to cisplatin and other chemotherapy drugs with effect in the treatment of human breast cancer. Hence, the data presented in our study are prerequisite to a forthcoming study on new clinical applications.

Naturally, there are drawbacks in our study that could be solved in future studies, including the small number and the short follow-up time of collected clinical samples for survival analysis. Therefore, the prognostic analysis of clinical data applied in our research did not uncover profound clinical value, where more prognostic clinical data need to be adopted. In conclusion, SERPINA3 can regulate the proliferation, migration and invasion of TNBC cells. In addition, EMT progression also shaped a corresponding plausible change, whilst the discovery of drug resistance to chemotherapy drugs was recognized under the overexpression of SERPINA3. Nevertheless, the clinical prognostic significance of which in breast tumors needs to be elaborated in an obviously larger number of patients with a longer follow-up period.

## Supplementary Information

Below is the link to the electronic supplementary material.

Supplementary file1 (PDF 177 KB)

Supplementary file2 (PDF 133 KB)

Supplementary file3 (PDF 479 KB)

Supplementary file4 (PDF 185 KB)

Supplementary file5 (PDF 230 KB)

## Data Availability

The datasets generated during and/or analyzed during the current study are available from the corresponding author on reasonable request.

## Abbreviations

BC: Breast cancer
CCK8: Cell counting kit 8 assay
EC: Endometrial carcinoma
EZH2: Enhancer of zeste homolog 2
EMT: Epithelial-mesenchymal transition
ER: Estrogen receptor
ECM: Extracellular matrix
FBS: Fetal bovine serum
GEO: Gene expression omnibus
GEPIA: Gene expression profling interactive analysis
GBM: Glioblastoma
HCC: Hepatocellular carcinoma
HR: Hormone receptor
HER2: Human epidermal growth factor receptor 2
OD: Optical density
PR: Progesterone receptor
q-PCR: Quantitative PCR
ROS: Reactive oxygen species
SERPINA3: Serpin peptidase inhibitor, clade A (alpha-1 antiproteinase, antitrypsin), member 3
TCGA: The cancer genome atlas
TNBC: Triple-negative breast cancer
TBST: Tris-buffered saline containing Tween-20
ZEB1: Zinc-finger E-box binding protein 1
