# Supplementary material for: Overexpression of SERPINA3 promotes tumor invasion and migration, epithelial-mesenchymal-transition in triple-negative breast cancer cells
Source: Breast Cancer. 2021 Feb 10;28(4):859–73. doi: 10.1007/s12282-021-01221-4 (PMC8213666; doi:10.1007/s12282-021-01221-4)
Supplement: Supplementary file 1 — Supplementary file1 (PDF 177 KB) [file 12282_2021_1221_MOESM1_ESM.pdf]

Overexpression of SERPINA3 promotes tumor invasion and migration, epithelial-mesenchymal-transition in triple negative breast cancer cells

Yingzi Zhang[1], JiaoTian[1], Chi Qu[1], Yang Peng[1], Jinwei Lei[1], Kang Li[1], Beige Zong[1], Lu Sun[1], Shengchun Liu[1]*

[1]Department of Endocrine Breast Surgery, The First Affiliated Hospital of Chongqing Medical University, 1 Yixueyuan Road, Yuanjiagang, Yuzhong district, Chongqing, China.

* Corresponding author: Shengchun Liu

E-mail addresses:

liushengchun1968@163.com

Author's details

Yingzi Zhang[1]: Email: zhangyingzi119@163.com

JiaoTian[1]: Email: 424045196@qq.com

Chi Qu[1]: Email: 565540717@qq.com

Yang Peng[1]: Email: pengyangpoop6@qq.com

Jinwei Lei[1]: Email: leijinweihenan@126.com

Kang Li[1]: Email: likang@stu.cqmu.edu.cn

Beige Zong[1]: Email: 504715943@qq.com

Lu Sun[1]: Email: 234952552@qq.com

Shengchun Liu[1]*: Email: liushengchun1968@163.com

Online Resource 1

A

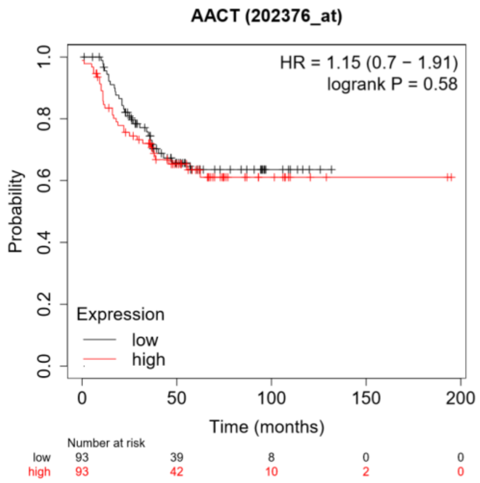

B

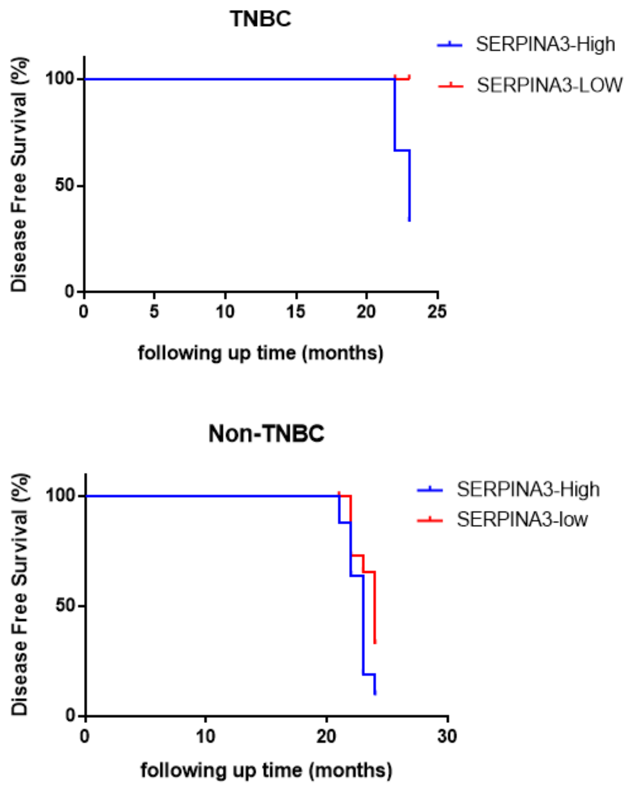

Survival analysis of human breast cancer patients.

(A) Kaplan–Meier overall survival analysis of human breast cancer patients with SERPINA3 expression

level from the TCGA database.

High expression of SERPINA3 was associated with poor prognosis of triple negative breast cancer patients revealed by data from GEPIA (p=0.58).

(B) Disease free survival analysis of different subtypes of clinical breast cancer patients (n=40). TNBC group: p=0.1573, Non-TNBC group: p=0.0349.
