# Supplementary material for: Overexpression of SERPINA3 promotes tumor invasion and migration, epithelial-mesenchymal-transition in triple-negative breast cancer cells
Source: Breast Cancer. 2021 Feb 10;28(4):859–73. doi: 10.1007/s12282-021-01221-4 (PMC8213666; doi:10.1007/s12282-021-01221-4)
Supplement: Supplementary file 3 — Supplementary file3 (PDF 479 KB) [file 12282_2021_1221_MOESM3_ESM.pdf]

Overexpression of SERPINA3 promotes tumor invasion and migration, epithelial-mesenchymal-transition in triple negative breast cancer cells

Yingzi Zhang[1], JiaoTian[1], Chi Qu[1], Yang Peng[1], Jinwei Lei[1], Kang Li[1], Beige Zong[1], Lu Sun[1], Shengchun Liu[1]*

[1]Department of Endocrine Breast Surgery, The First Affiliated Hospital of Chongqing Medical University, 1 Yixueyuan Road, Yuanjiagang, Yuzhong district, Chongqing, China.

* Corresponding author: Shengchun Liu

E-mail addresses:

liushengchun1968@163.com

Author's details

Yingzi Zhang[1]: Email: zhangyingzi119@163.com

JiaoTian[1]: Email: 424045196@qq.com

Chi Qu[1]: Email: 565540717@qq.com

Yang Peng[1]: Email: pengyangpoop6@qq.com

Jinwei Lei[1]: Email: leijinweihenan@126.com

Kang Li[1]: Email: likang@stu.cqmu.edu.cn

Beige Zong[1]: Email: 504715943@qq.com

Lu Sun[1]: Email: 234952552@qq.com

Shengchun Liu[1]*: Email: liushengchun1968@163.com

Online Resource 3

A

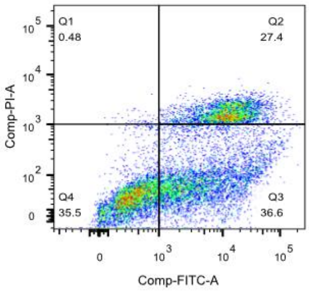

MDA-MB2-231
SERPINA3-VE

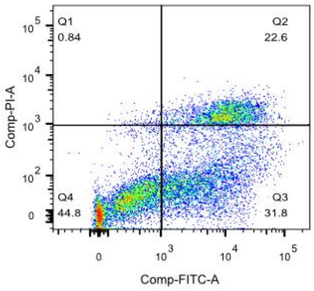

MDA-MB-231
SERPINA3-VE

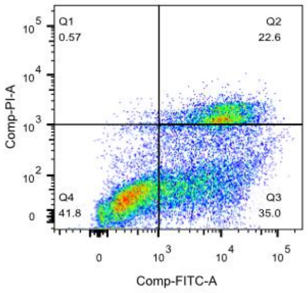

MDA-MB-231
SERPINA3-OE

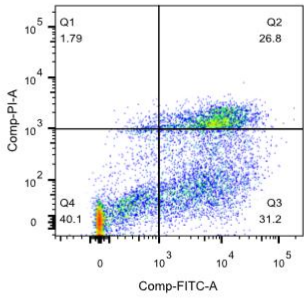

MDA-MB-2311
SERPINA3-OE

B

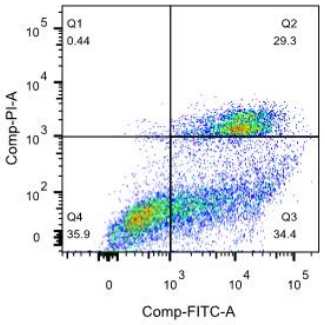

SERPINA3-VE01

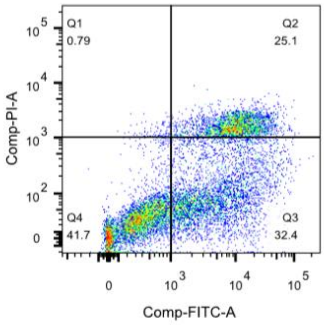

SERPINA3-VE02

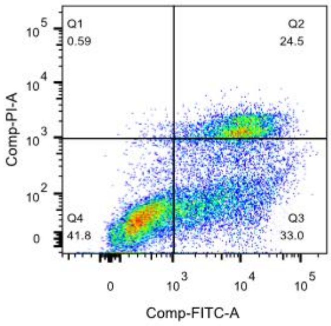

SERPINA3-OE01

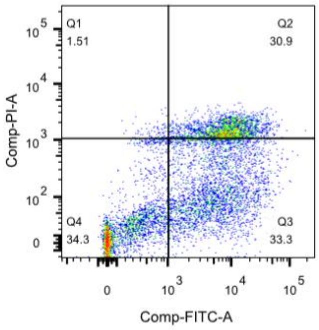

SERPINA3-OE02

C

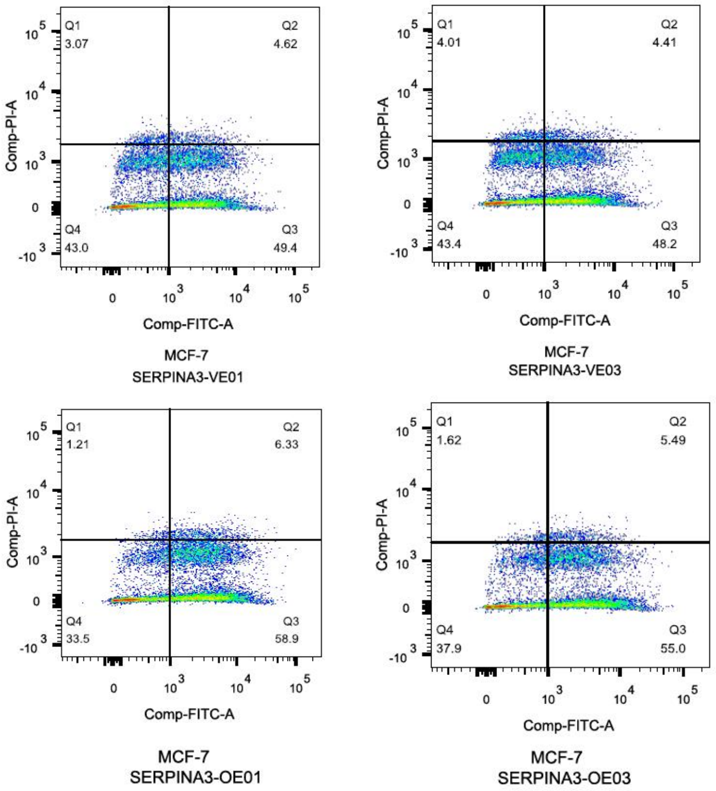
Q1
3.07
Q2
4.62
Q4
43.0
Q3
49.4
Comp-PI-A
Comp-FITC-A
MCF-7
SERPINA3-VE01
Q1
4.01
Q2
4.41
Q4
43.4
Q3
48.2
Comp-PI-A
Comp-FITC-A
MCF-7
SERPINA3-VE03
Q1
1.21
Q2
6.33
Q4
33.5
Q3
58.9
Comp-PI-A
Comp-FITC-A
MCF-7
SERPINA3-OE01
Q1
1.62
Q2
5.49
Q4
37.9
Q3
55.0
Comp-PI-A
Comp-FITC-A
MCF-7
SERPINA3-OE03

D

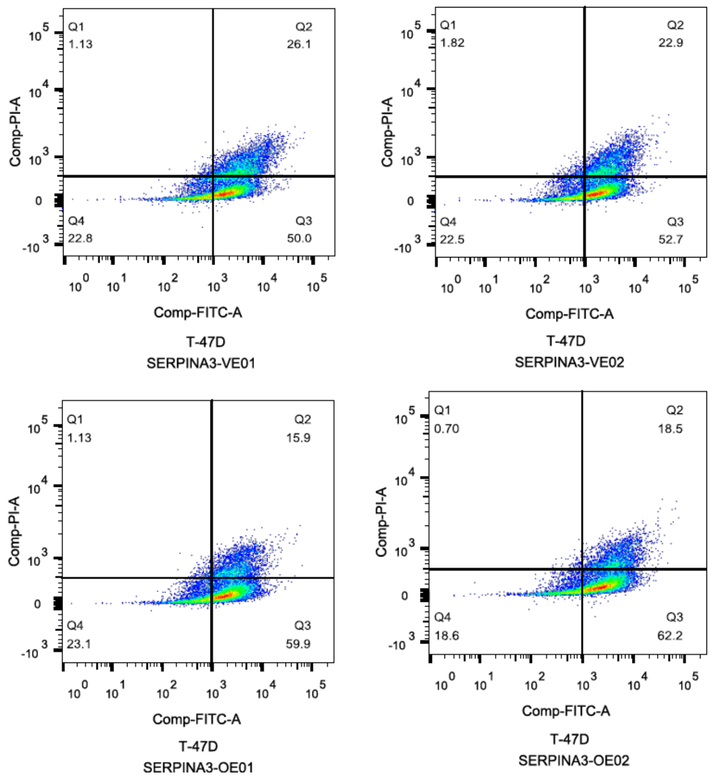
Q1
1.13
Q2
26.1
Q4
22.8
Q3
50.0
Comp-PI-A
Comp-FITC-A
T-47D
SERPINA3-VE01
Q1
1.82
Q2
22.9
Q4
22.5
Q3
52.7
Comp-PI-A
Comp-FITC-A
T-47D
SERPINA3-VE02
Q1
1.13
Q2
15.9
Q4
23.1
Q3
59.9
Comp-PI-A
Comp-FITC-A
T-47D
SERPINA3-OE01
Q1
0.70
Q2
18.5
Q4
18.6
Q3
62.2
Comp-PI-A
Comp-FITC-A
T-47D
SERPINA3-OE02

SERPINA3 influenced on TNBC cell apoptosis

a, b Flow cytometric assay was employed to analyze the cell apoptosis of TNBC cells

The percentage of cells in the early apoptosis phase was not evidently decreased, compared with the control group in MDA-MB-231 and MDA-MB-436.

c, d Flow cytometric assay was employed to analyze the cell apoptosis of non-TNBC cells

The percentage of cells in the early apoptosis phase was not evidently decreased either, compared with the control group in MCF-7 and T-47D.
