# Supplementary material for: Overexpression of SERPINA3 promotes tumor invasion and migration, epithelial-mesenchymal-transition in triple-negative breast cancer cells
Source: Breast Cancer. 2021 Feb 10;28(4):859–73. doi: 10.1007/s12282-021-01221-4 (PMC8213666; doi:10.1007/s12282-021-01221-4)
Supplement: Supplementary file 5 — Supplementary file5 (PDF 230 KB) [file 12282_2021_1221_MOESM5_ESM.pdf]

Overexpression of SERPINA3 promotes tumor invasion and migration, epithelial-mesenchymal-transition in triple negative breast cancer cells

Yingzi Zhang[1], JiaoTian[1], Chi Qu[1], Yang Peng[1], Jinwei Lei[1], Kang Li[1], Beige Zong[1], Lu Sun[1], Shengchun Liu[1]*

[1]Department of Endocrine Breast Surgery, The First Affiliated Hospital of Chongqing Medical University, 1 Yixueyuan Road, Yuanjiagang, Yuzhong district, Chongqing, China.

* Corresponding author: Shengchun Liu

E-mail addresses:

liushengchun1968@163.com

Author's details

Yingzi Zhang[1]: Email: zhangyingzi119@163.com

JiaoTian[1]: Email: 424045196@qq.com

Chi Qu[1]: Email: 565540717@qq.com

Yang Peng[1]: Email: pengyangpoop6@qq.com

Jinwei Lei[1]: Email: leijinweihenan@126.com

Kang Li[1]: Email: likang@stu.cqmu.edu.cn

Beige Zong[1]: Email: 504715943@qq.com

Lu Sun[1]: Email: 234952552@qq.com

Shengchun Liu[1]*: Email: liushengchun1968@163.com

Online Resource 5

A

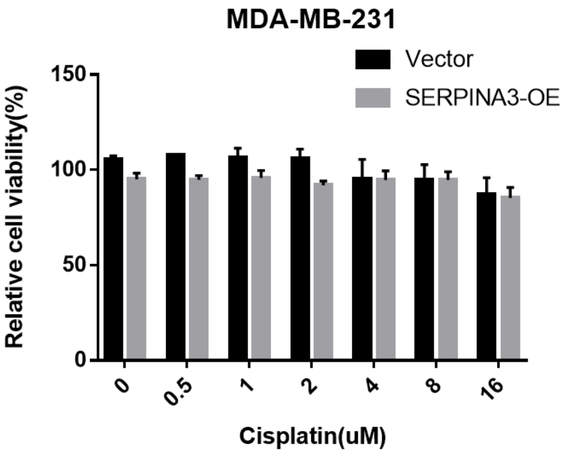

B

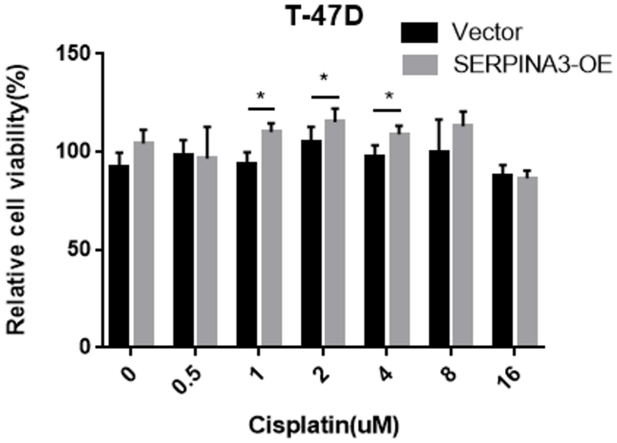

Overexpression of SERPINA3 influenced on the cell viability of BC cells with treatment of cisplatin.

a, b Comparation of cell viability of the SERPINA3-OE group in MDA-MB-231 and T-47D cells treated with a series of concentrations of cisplatin (uM) for 48 h and the control group (*p<0.05).
